# Tbx Protein Level Critical for Clock-Mediated Somite Positioning Is Regulated through Interaction between Tbx and Ripply

**DOI:** 10.1371/journal.pone.0107928

**Published:** 2014-09-26

**Authors:** Chimwar Wanglar, Jun Takahashi, Taijiro Yabe, Shinji Takada

**Affiliations:** 1 Okazaki Institute for Integrative Bioscience and National Institute for Basic Biology, National Institutes of Natural Sciences, Okazaki, Aichi, Japan; 2 The Graduate University for Advanced Studies (SOKENDAI), Okazaki, Aichi, Japan; Instituto Gulbenkian de Ciência, Portugal

## Abstract

Somitogenesis in vertebrates is a complex and dynamic process involving many sequences of events generated from the segmentation clock. Previous studies with mouse embryos revealed that the presumptive somite boundary is periodically created at the anterior border of the expression domain of Tbx6 protein. *Ripply1* and *Ripply2* are required for the determination of the Tbx6 protein border, but the mechanism by which this Tbx6 domain is regulated remains unclear. Furthermore, since zebrafish and frog *Ripplys* are known to be able to suppress Tbx6 function at the transcription level, it is also unclear whether Ripply-mediated mechanism of Tbx6 regulation is conserved among different species. Here, we tested the generality of Tbx6 protein-mediated process in somite segmentation by using zebrafish and further examined the mechanism of regulation of Tbx6 protein. By utilizing an antibody against zebrafish Tbx6/Fss, previously referred to as Tbx24, we found that the anterior border of Tbx6 domain coincided with the presumptive intersomitic boundary even in the zebrafish and it shifted dynamically during 1 cycle of segmentation. Consistent with the findings in mice, the *tbx6* mRNA domain was located far anterior to its protein domain, indicating the possibility of posttranscriptional regulation. When both *ripply1/2* were knockdown, the Tbx6 domain was anteriorly expanded. We further directly demonstrated that Ripply could reduce the expression level of Tbx6 protein depending on physical interaction between Ripply and Tbx6. Moreover, the onset of *ripply1* and *ripply2* expression occurred after reduction of FGF signaling at the anterior PSM, but this expression initiated much earlier on treatment with SU5402, a chemical inhibitor of FGF signaling. These results strongly suggest that Ripply is a direct regulator of the Tbx6 protein level for the establishment of intersomitic boundaries and mediates a reduction in FGF signaling for the positioning of the presumptive intersomitic boundary in the PSM.

## Introduction

Somites, which are segmental epithelial blocks located symmetrically on either side of the neural tube, are periodically generated in an anterior to posterior manner from their precursors, known as the presomitic mesoderm (PSM), which is located posterior to the newly formed somites. This periodic generation is achieved by a complex and dynamic mechanism operating in the PSM [1]–[5]. First, a molecular clock, the so-called segmentation clock, creates oscillatory expression of particular genes, *hairy* and other *notch*-related genes, in the posterior PSM. The period of oscillation is almost consistent during somitogenesis, for instance, 120 min in the mouse and 20 to 30 min in the zebrafish. Because the phase of oscillation among PSM cells is gradually delayed in a posterior-to-anterior direction, a wave of the oscillation appears to move in a posterior-to-anterior fashion. This oscillatory gene expression subsequently results in periodical generation of morphologically segmented somites.

The segmental pattern of somites is primarily defined by positioning of presumptive intersomitic boundaries. The position of each boundary is repeatedly established in an anterior-to-posterior order in accordance with posterior elongation of body length. Furthermore, the time interval of this boundary formation is coupled with the time period of the segmentation clock. Thus, during the process of the boundary formation, the oscillatory gene expression is converted into a spatial pattern with periodicity. A number of transcription factors and cell-to-cell signaling molecules are involved in this conversion [4], [6]–[13]. For instance, the oscillatory changes in FGF and Notch signalings determine the onset of expression of *Mesp2*, a transcription factor involved in the spatial patterning of somites, at the anterior PSM in the mouse embryos [12], [14]. Then, *Mesp2* expression defines the spatial pattern of Tbx6, which plays another critical role in the positioning of the segmentation boundary [14]–[17]. The presumptive segmentation boundary is generated at the anterior border of the expression domain of Tbx6 protein, which is posteriorly shifted by 1 segment length during the time period of 1 segmentation cycle [14]. Conversely, Tbx6 is indispensable for the PSM expression of *Mesp2*, indicating that Tbx6 and Mesp2 are mutually regulated. This feedback loop between Mesp2 and Tbx6 appears to regulate the periodical shift of the anterior border of the expression domain of Tbx6 protein, which is referred to as “Tbx6 domain” hereinafter [5].

Importantly, the anterior border of the Tbx6 domain is not consistent with that of *Tbx6* mRNA, but rather regulated by a proteasome-mediated mechanism [14]. Although the molecules directly executing this proteolysis are still unclear, studies with knockout mice indicate that *Ripply1* and *Ripply2*, as well as *Mesp2*, are required for the down-regulation of Tbx6 proteins [18]–[20]. In addition, considering that the expression of *Ripply1* and *Ripply2* in the PSM is lost in *Mesp2*-deficient mouse embryos, we previously proposed the following model: *Mesp2*, whose expression is activated in the most anterior part of the Tbx6 domain, causes retreat of the Tbx6 domain through activation of *Ripply1* and *Ripply2* expression, and the retreated Tbx6 subsequently defines the next segmentation border and *Mesp2* expression [20]. However, this model must be validated in several different ways, one for instance, is by elucidating whether Ripply1 and/or Ripply2 can actually suppress the protein level of Tbx6.

Tbx6 appears to play an essential role in the boundary formation in other animals. For instance, zebrafish defective for *tbx6/fss*, previously referred to as *tbx24*, exhibit defective boundary formation as in the case of its mouse counterpart [21], [22]. However, in contrast to the analysis with mouse mutants, previous studies with zebrafish and Xenopus Ripply suggested another function of Ripply in the regulation of Tbx6 [23]–[27]. In cultured cells, Ripply1, Ripply2, and Ripply3 suppress the transcriptional activation mediated by Tbx6. Ripply1 associates with Tbx6 and converts it to a repressor. A mutant form of Ripply1, defective in association with Tbx6, lacks this activity in zebrafish embryos. These results indicate that the intrinsic transcriptional property of T-box proteins is also controlled by Ripply family proteins, which act as specific adaptors that recruit the global co-repressor Groucho/TLE to T-box proteins in this context. Thus, it is still unclear whether Ripply regulates Tbx6 proteins at the protein level even in other animals except the mouse.

For a better understanding of the mechanism of Tbx6-mediated patterning of somites, in this present study, we examined whether the expression pattern of Tbx6 proteins correlate with the positioning of intersomitic boundaries in the zebrafish by generating antibody specific for zebrafish Tbx6, and whether zebrafish *ripply* is required for reduction of Tbx6 proteins. Since these experiments showed that *ripply*-dependent regulation of Tbx6 protein in the positioning of somite boundary was significantly common in the zebrafish and the mouse, we further examined the ability of Ripply to reduce the level of Tbx6 proteins by co-injecting *Tbx6* mRNA and *Ripply* mRNA into zebrafish eggs. Finally, we examined the relationship between *ripply* expression and FGF signaling, another key factor in the positioning of somite boundaries. These results strongly suggest that Ripply is a critical regulator of the Tbx6 protein level in the establishment of intersomitic boundaries and that this mechanism is conserved among vertebrates.

## Materials and Methods

### Ethics Statement

This study was performed in accordance with the Guidelines for Animal Experimentation of National Institutes of Natural Sciences, with approval of the Institutional Animal Care and Use Committee (IACAC) of National Institutes of Natural Sciences, and all efforts were made to minimize suffering during experimental procedures.

### Fish

Zebrafish were maintained at 28°C on a 14-h light/10-h dark cycle. All studies on wild- type fish were performed by using the TL2 inbred line [28].

### *In Situ* Hybridization

Whole-mount *in situ* hybridization of zebrafish embryos was carried out according to the protocol previously described [29]. Probes were synthesized for *mesp-aa/ba* [30], *tbx6* [22], *ripply1/2* [23], by using a standard protocol. The fragments of *mesp-ab/bb* were amplified by PCR and cloned into pBS-SK+ or pGEM-T easy vector respectively, to synthesize the RNA probe. For fluorescence *in situ* hybridization, the probes were labeled with digoxigenin and color was detected by using TSA Plus-Fluorescein Solution [31].

### Antibody preparation and whole mount immunostaining

For immunostaining of zebrafish Tbx6 proteins, we generated anti-rabbit antibody against zebrafish Tbx6. The immunogen was prepared from *E. coli*. expressing a fragment of the zebrafish Tbx6, ranging from the 561st to the 874th position in its amino-acid sequence. Purified proteins electroeluted from poly-acrylamide gel were used to immunize 2 rabbits. After 7 injections of the purified proteins, sera (#1 and #2) were recovered from the rabbits; and their reactivity and specificity were assessed by Western blotting (Figure S1). Whole mount immunostaining was conducted using one of the antisera (#1) at a dilution of 1∶200 in 2%BSA-PBS containing 0.1% Triton-x100, with incubation for 48 hrs at 4°C and detected with alexa fluor-555 anti-rabbit antibody (Invitrogen). For quantification of the expression levels of *tbx6* mRNA and Tbx6 protein in the PSM, signal intensity was measured by ImageJ software (National Institute of Health) and background was subtracted. Obtained intensity values were normalized to a range between 0 and 1. Immunostatining with anti-pErk (Sigma) was performed according to the protocol by [32]. To compare Tbx6 protein pattern with *tbx6, her1, mesp-ab, mesp-ba, ripply1*, and *ripply2* mRNAs, immunostaining was performed after *in situ* hybridization.

### Antisense MO injection

The sequences of morpholinos used in this study were the following: *her1* MO 5′-GACTTGCCATTTTTGGAGTAACCAT-3′ and *her7* MO 5′-TTTCAGTCTGTGCCAGGATTTTCA-3′ [34]; *ripply1* MO1, 5′-CATCGTCACTGTGTTTTTCGTTTTG-3′ and 5mis-*ripply1* MO1, 5′-CtTCcTCAgTGTcTTTTTCcTTTTG-3′ [23]; *ripply2* MO1, 5′- TCGTGAAAGTGATGTTCTCCATAGT-3′ [35]; 5mis-*ripply2* MO1, 5′-AGTCATCTTCTGCATAGTCTCGATG-3′ and *ripply2* MO2, AGTGATGTTCTCCATAGTGTCCATG. Neither of the *ripply2* morpholinos gave a phenotypic change when injected alone. We continued the experiments with the *ripply2* MO2. Embryos were injected at the 1-cell stage and fixed at 8 somite stage for overnight at 4°C with 4%PFA. One ng of *ripply1* morpholino; 2 ng of *ripply2* morpholino and 1∶2 of *ripply1*: *ripply2* MOs were injected. *her1* and *her7* morpholinos were each diluted to 4 mg/ml working solution and co-injected at a ratio of 1∶1. The morphants exhibit weak boundaries as described. Morpholinos were diluted in sterile milliQ water and supplemented with 0.1% Phenol red (SIGMA) in 0.1 M KCl (Nacalai Tesque) for injection.

### mRNA injection and preparation of cell lysates for SDS PAGE

Capped mRNAs were transcribed from linearized pCS2+zRipply1-Myc, pCS2MT+zRipply1-6Myc, pCS2MT+zRipply1mutFPVQ-6Myc, pCS2+mRipply2-Myc, pCS2+mRipply2mutFPIQ-Myc, pCS2+mTbx6-Flag, pCS2+mBrachyury-Flag, and pCS2+GFP by using an mMessage mMachine Sp6 kit (Ambion). Zebrafish *tbx6-Flag* mRNA was synthesized from a template DNA amplified by PCR. mRNAs were injected, at the desired concentrations, at the 1-cell stage and the eggs were harvested after 6 hrs of incubation at 28.5°C. After careful dechorionation, the intact eggs were collected into 1.5-ml tubes (20 eggs/tube) on ice. Then the eggs were triturated with a 200-µl micropipette having a broken tip. Next, 2x SDS PAGE buffer (2 µl/embryo) was added to the pellet and the cells were vortexed. In some experiments, 200 µl of protease inhibitor cocktail (Nacalai Tesque) was added before the trituration, and the eggs were centrifuged twice at 1000 rpm at 4°C for 1 min each after the trituration and the supernatant was carefully removed. After the tubes had been immersed in liquid nitrogen, the samples were then either stored at −80°C or continued by boiling for 10 min at 95°C before being loaded into the PAGE gel [36]. Western blotting was performed according to a standard procedure with anti-zebrafish Tbx6 rabbit polyclonal antibody (#2), anti-Myc mouse monoclonal antibody, clone 4A6 (Upstate, 05-724), anti-Myc rabbit polyclonal (Abcam, ab9106), anti-GFP rabbit polyclonal (MBL, 598) and anti-Flag rabbit polyclonal antibody (Sigma, F7425).

### Immunoprecipitation

Whole cell lysates were prepared from 293T or COS7 cells transfected with pCS2+zTbx6-Flag, pCS2MT+zRipply1-6Myc, pCS2MT+zRipply1mutFPVQ-6Myc, pCS2+mTbx6-Flag, pCS2+mRipply2-Myc, pCS2+mRipply2mutFPIQ-Myc, or pCS2+ expression vectors accordingly. The lysates were pre-cleared by passing over Protein G resin bed (GE Healthcare) for 2 hrs at 4°C to eliminate any unspecific binding. The solution was then incubated with anti-FLAG M2 resin (SIGMA) for 3 hrs at 4°C to allow antibody-antigen complexes to form. The precipitated complex was washed several times, and the proteins were collected in 2x SDS sample buffer and separated by SDS PAGE. Western blotting was conducted and the proteins were detected by anti rabbit polyclonal antibody against zebrafish Tbx6 (#2) or rabbit polyclonal anti-Myc (Abcam, ab9106) antibodies accordingly.

### Chemical treatment of zebrafish embryos

DAPT, a Notch inhibitor, was used as described earlier [37]. Chorions were removed from eggs at around the 75% epiboly stage, and the embryos were incubated with 100 µM DAPT at 28.5°C and then fixed at 10 somite stage. SU5402 was used as described previously [7]. Embryos were dechorionated and treated with 0.4 mM SU5402 at 2 somite stage for 8 min. After thorough washing, they were incubated at 28.5°C and then fixed at 6 somite stage for overnight at 4°C with 4% PFA. Some of the embryos were fixed just after SU5402 treatment and analyzed.

Statistical analysis was performed by the following procedure. Distance was measured from the chordo neural hinge to the anterior border of the posteriormost band of the *ripply1* mRNA expression, for both the control and the SU5402 treated embryos expressing *ripply1* mRNA, by ImageJ. Standard deviation for each measurement was calculated and a student's t test was performed for two samples with unequal variances. A p<0.05 value was obtained.

## Results

### Periodical change in the anterior border of the Tbx6 domain in zebrafish embryos

**Figure 1:**
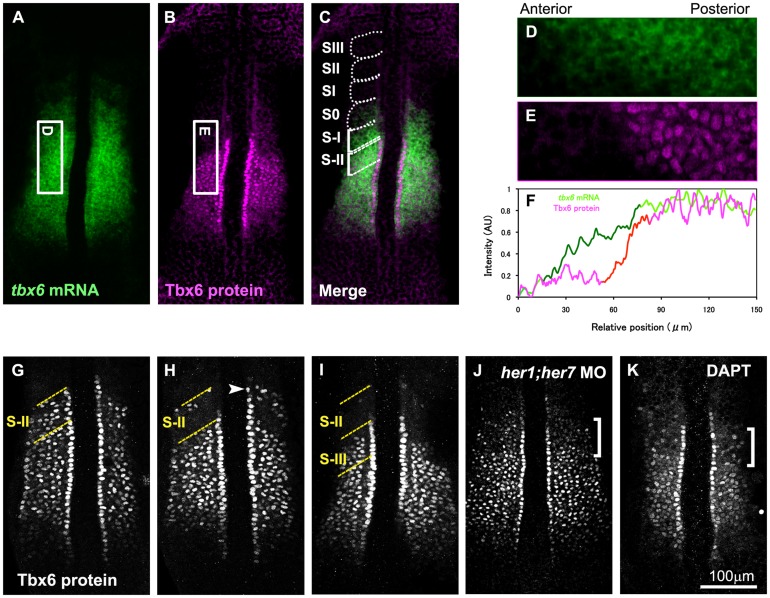
Periodic expression of Tbx6 protein and posttranscriptional regulation of its anterior border. (A-C) *In situ* hybridization with *tbx6* probe (A) and immunostaining with anti-Tbx6 antibody (B) were performed using zebrafish embryos at the 8 somite stage (*n* = 15). Merged image (C) combined (A) and (B) is also shown. Zebrafish *tbx6* mRNA is expressed broadly throughout the anterior PSM. At the same time, the Tbx6 protein is also expressed throughout the anterior PSM, however, its anterior border is restricted far posterior to the anterior border of the mRNA. The position of each segmental unit is also indicated from SIII to S-II. (D-F) Quantitative analysis of *tbx6* mRNA and protein in the PSM. Intensity of *tbx6* mRNA signals in a boxed area in the PSM (D; the boxed area shown in (A) is indicated by 90° rotation) and protein signals in the corresponding area (E; the boxed area shown in (B) is indicated by 90° rotation) was scanned and indicated by green and magenta lines, respectively in (F). While *tbx6* mRNA is gradually decreased in the anterior region (shown by dark green line), Tbx6 protein level is abruptly decreased (shown by red line). Anterior is left and posterior is right. (G-I) Indication of 3 typical patterns of embryos stained with anti-Tbx6 antibody. Embryos were observed at 8 somite stage. Comparative analysis with *her1* mRNA expression shown in Figure 2 indicates that the anterior border of the Tbx6 protein follows a phase of periodic change. After a long core domain of Tbx6 proteins is generated (G), an anterior part of the Tbx6 protein domain was eliminated, resulting in appearance of the upper band, which is indicated by an arrowhead (H), then this upper band disappeared, resulting in a short Tbx6 domain (I). Out of a total of 154 embryos examined, around 40% of them showed (G), 35% showed (H), 25% showed (I) type of expression pattern. (J) A 10 somite stage embryo injected with both *her1* and *her7* specific antisense morpholino oligos was stained with anti-Tbx6 antibody. The defects were observed in 97.5% of the injected embryos (*n* = 40). (K) A 10 somite stage embryo treated with DAPT, a Notch inhibitor, was stained with anti-Tbx6 antibody. The defects were observed in all of the embryos treated with DAPT (*n* = 22). Pattern of Tbx6 proteins was disturbed in anterior area indicated by a bracket (J, K). The yellow dotted lines indicate S-II (G, H) and S-II and S-III (I) regions.

As a first step toward understanding the regulation of zebrafish Tbx6 protein expression during somitogenesis, we generated anti-Tbx6 antibody suitable for immunohistochemistry and observed its localization in the PSM of zebrafish embryos at around the 8-somite stage. As predicted from its mRNA pattern, zebrafish Tbx6 proteins were broadly expressed in the anterior PSM (Figures 1A-1C). However, similar to its counterpart in the mouse, the anterior limit of the Tbx6 protein domain was shifted posterior to that of its mRNA domain, forming a clear border; whereas the posterior limit was almost identical between these 2 domains (Figures 1D-1F). This result suggests that the anterior border of the Tbx6 protein was regulated post-transcriptionally as in the case of the mouse [14].

**Figure 2:**
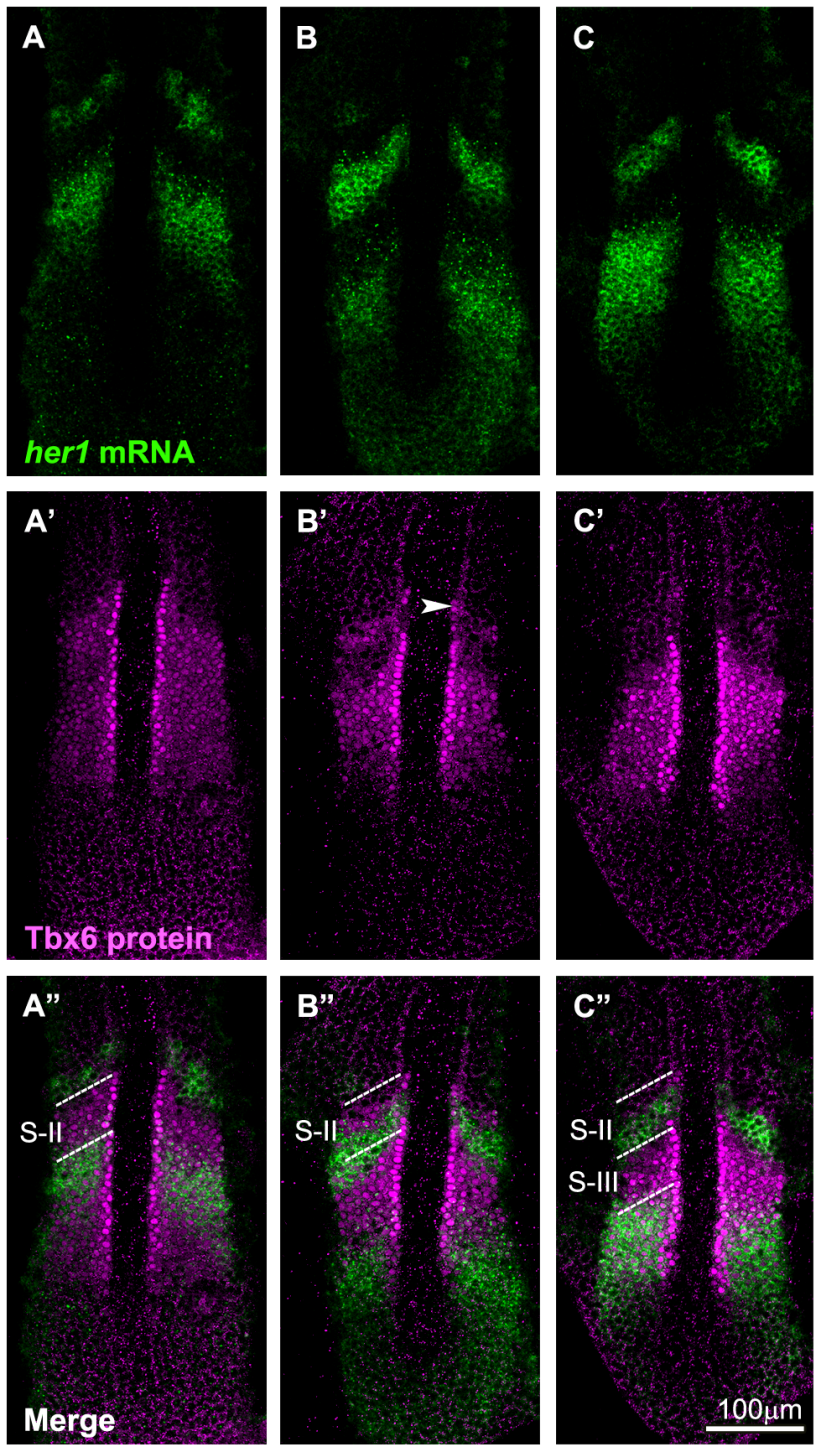
Comparative analysis of the anterior border of the Tbx6 domain with expression of *her1*. Spatial pattern of the Tbx6 protein (A′-C′; magenta) is compared with those of *her1* mRNA (A-C; green) at 3 different phases of segmentation cycle at around the 8 somite stage. Merged images are also indicated (A″-C″). According to the general nomenclature [39], the phases shown in A, B, and C appear to correspond to the phase III, I, and II, respectively. (B-B″) Anterior Tbx6 starts to disappear with some remains (the upper band: arrowhead) (B′). (C-C″) The upper band of Tbx6 disappears and the next Tbx6 anterior border shifts posteriorly. (A-A″) The core domain was extended posteriorly. Out of a total of 42 embryos examined, around 35% of them showed A type, 41% showed B type, 24% showed C type of expression. The dotted lines indicate S-II (A″, B″) and S-II, S-III (C″) regions.

However, unlike mouse Tbx6 proteins, an additional distinct band of zebrafish Tbx6 protein was detected anterior to this broad domain in 35% of stained embryos (Figure 1H). We refer to this distinct band as “upper band“ and the broad protein domain as the “core domain” hereinafter. Of note, the length of the core domain along the A-P axis changed within the length of 1 segment (Figures 1G-1I). To examine whether the patterns of Tbx6 proteins correlated with the phases of oscillation, we examined the expression pattern of *her1*, a zebrafish gene related to *hairy* and *enhancer of split*, [30], [38] in the same embryos and identified the phase of the oscillation cycle (Figures 2A-2C; [39]. In the PSM of zebrafish embryos, *her1* is expressed in several distinct domains along the anterior-posterior axis. During a segmentation cycle, the most posterior expression is initially observed in broad area of the posterior PSM (phase I), then this expression becomes more discrete and gradually shifts to the anterior direction (phase II and III). The comparative analysis revealed that a long core domain, without the upper band, of Tbx6 protein was observed in phase III (Figure 2A′). At this phase, the anterior limit of the Tbx6 protein domain coincided with B-II, the boundary between presumptive somite S-II and S-III. The upper band emerged from late phase III to early phase I (Figure 2B′), then this upper band diminished (Figure 2C′) and the core domain, whose anterior limit now coincided with B-III, gradually extended to the posterior direction by 1 segment length during phase II and III. This means elimination of Tbx6 proteins takes place in a two-step fashion; it started in the anterior part of the core domain, except in the most anterior part of it, and then proteins persisting in the upper band subsequently disappeared. Taking into consideration that spatial pattern of *tbx6* mRNA remained continuous without showing any upper band during a single segmentation cycle, this dynamic change in Tbx6 proteins shows the importance of post-transcriptional regulation in the spatial patterning of the Tbx6 domain.

**Figure 3:**
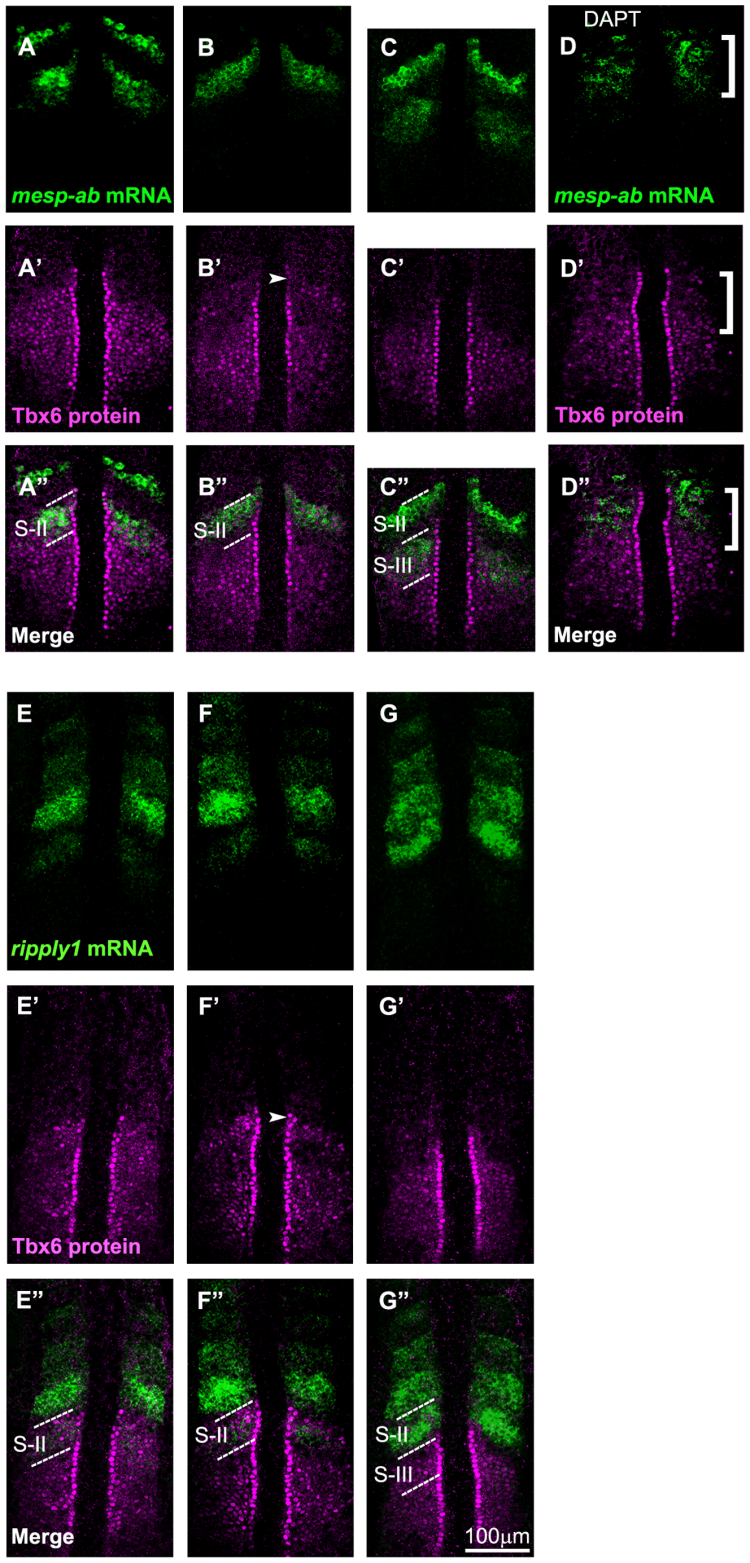
Comparative analysis of the anterior border of the Tbx6 domain with expression of *mesp* and *ripply*. Spatial pattern of the Tbx6 protein (magenta) is compared with those (green) of *mesp-ab* mRNA (A-D) and *ripply1* mRNA (E-G) at 3 different phases of segmentation cycle (the phases in embryos shown in A, B, or C are identical to those shown in E, F, or G, respectively) at around the 8 somite stage. Tbx6 pattern was also compared with *mesp-ab* mRNA in embryos treated with DAPT (D). Merged images are indicated (A″-G″). Out of a total of 59 embryos examined, around 46.5% of them showed A and E phase, 27% showed B and F phase, 26.5% showed C and G phase type of expression. Note that the anterior limit of newly expressed, or most posterior, *mesp-ab* band coincided with the anterior border of the Tbx6 core domain (A-A″). Then, this expression coincided with the upper band of Tbx6 when elimination of the anterior Tbx6 domain started (B-B″). On the other hand, new *ripply1* expression emerged within the anterior part of Tbx6 domain (E-E″) and the Tbx6 domain starts to vanish in area where *ripply1* was expressed (F-F″). In (D), the defects were observed in all of the embryos treated with DAPT (*n* = 22). Patterns of Tbx6 proteins and *mesp-ab* mRNA were disturbed in anterior area indicated by a bracket. The dotted lines indicate S-II (A″, B″, E″, F″) and, S-II and S-III (C″, G″) regions.

To examine relationship between Tbx6 protein pattern and the prospective segmentation border, we next compared the spatial pattern of zebrafish Tbx6 proteins with that of mRNA of *mesp* genes. The zebrafish possesses at least 4 *mesp* genes; 2 recently identified ones, *mesp-ab* and *mesp-bb* [40], in addition to *mesp-aa* and *mesp-ba*, previously referred to as *mesp-a and mesp-b*, respectively. These 4 *mesp* genes are expressed in the anterior PSM in a similar fashion. For instance, the anterior expression border of these 4 *mesp* genes coincides with the prospective segmentation boundaries in the anterior PSM [30], [40]. The onset of *mesp-ab* and *mesp-ba* expression, which occurred at the level of S-II, was observed at the most anterior region of the core domain of the Tbx6 protein (Figures 3A-3C, and Figure S2). Thus, as in the case of mouse embryos, the anterior border of the Tbx6 core domain basically coincided with the prospective segmentation boundary even in the zebrafish, suggesting that the mechanism governing Tbx6 protein-mediated segmentation is conserved between mouse and zebrafish.

If this is true, the anterior border of the Tbx6 domain should be perturbed in embryos in which formation of the intersomitic boundary is defective. In the zebrafish, *her1* and *her7*, encoding transcriprional repressors crucial for establishment of the segmentation clock, are required for proper formation of somite boundaries. We found that the anterior border of the Tbx6 protein domain was not clear in embryos injected with antisense morpholino oligos specific both for *her1* and *her7* (Figure 1J). In addition, Notch-defective embryos show impaired segmentation due to de-synchronization of oscillation among PSM cells, resulting in a change in the expression patterns of several *mesp* genes into “salt-and-pepper” ones [41]. We also observed that the anterior border of the Tbx6 proteins was actually disturbed in embryos treated with DAPT, N-[N-(3,5-difluorophenyacetyl)-L-alanyl]-S-phenyl glycine t-butylester, which inhibits γ-secretase and widely used as a Notch pathway inhibitor, supporting the correlation between the anterior border of the Tbx6 domain and the prospective segmentation boundary (Figures 1K and 3D).

### *ripply1* and *ripply2* are required for proper positioning of Tbx6 domain in zebrafish embryos

We next compared expression of *ripply1* and *ripply2* with the Tbx6 domain (Figures 3D-3F, and Figure S3). The earliest, or the most posterior, expression occurred at the S-II level in the anterior part of the core domain of the Tbx6 domain. After these earliest signs of *ripply1* and *ripply2* mRNA expression, Tbx6 protein started to become reduced in anterior part of the core domain. Since the region where Tbx6 proteins became reduced well coincided with the area where *ripply1* and *ripply2* had been expressed in the core domain, these Ripplys appeared to function to reduce expression of the Tbx6 protein.

**Figure 4:**
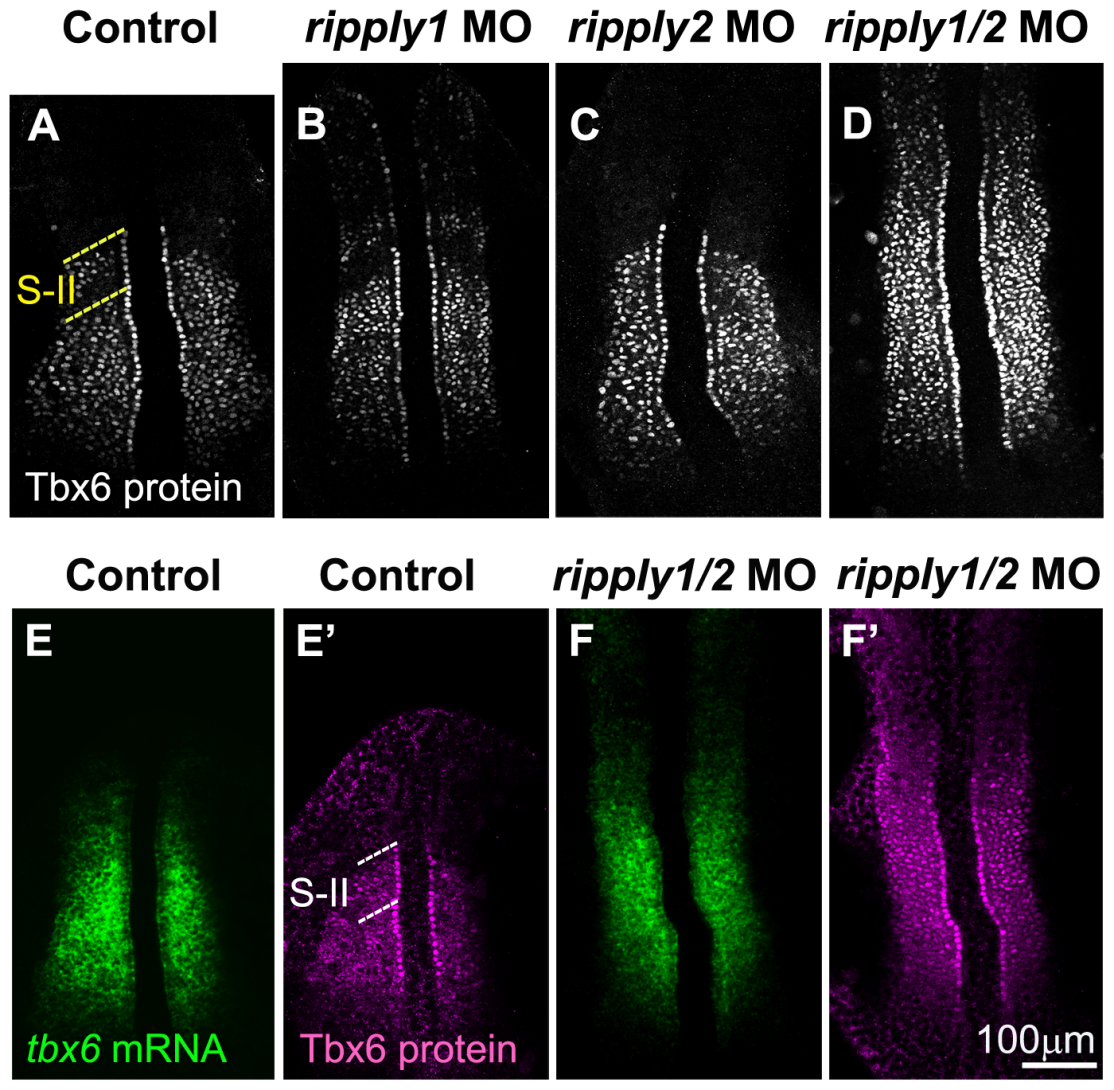
Proper positioning of Tbx6 domain depends on *ripply*. (A-D) Patterns of Tbx6 protein at the 8 somite stage in control (n = 20) (A), *ripply1* morphant (n = 25) (B), *ripply2* morphant (n = 20) (C) and *ripply1/ripply2* double morphant (n = 30) (D). Comparison of Tbx6 protein (E′, F′) with its mRNA (E, F) patterns in control (E, E′) and *ripply1/ripply2* double morphant (F, F′). *ripply1* morphants show graded expansion of Tbx6 protein anteriorly (B) but *ripply2* morphants (C) show no significant difference from control embryos (A). Double knockdown of *ripply1* and *ripply2* show strong expansion of Tbx6 protein anteriorly (D). In the double morphants, *tbx6* mRNA is also anteriorly expanded to some level, but not so significantly as Tbx6 protein (F, F′). A total of 20 injected embryos were observed for each injection. While *ripply2* morphant appeared similar to control embryos in Tbx6 protein pattern, 100% of the *ripply1* morphants and 100% of the *ripply1* and *ripply2* double morphants displayed anterior expansion of Tbx6 protein shown in (B) and (D) respectively. The dotted lines indicate S-II (A, E′) region.

To validate our theory that *ripply1* and *ripply2* actually play a role in reducing the Tbx6 protein level in zebrafish embryos, we examined the spatial pattern of Tbx6 proteins in *ripply1* and/or *ripply2*-deficient embryos (Figure 4). Injection of antisense morpholino oligos specific for zebrafish *ripply1* and *ripply2* caused severe expansion of the Tbx6 domain in zebrafish embryos (Figure 4D). This expansion was certainly, or at least to some extent, a result of posttranscriptional dysregulation, since the *tbx6* mRNA domain was not so severely, but only slightly expanded as compared to the protein domain in *ripply1/ripply2* double-deficient embryos (Figures 4E, 4E′, 4F, and 4F′). On the other hand, *ripply1* single morphants exhibited less severe expansion of the Tbx6 domain (Figure 4B); whereas this domain looked normal in *ripply2* single morphants (Figure 4C), indicating a redundant role between these 2 ripplys in the regulation of Tbx6 protein expression. Consistent with these results, *ripply1/ripply2* double-deficient embryos, as well as *ripply1* single morphants, exhibited no segmentation boundary; whereas *ripply2* single morphants seemed normal in the morphology of their somites. Therefore, the 2 ripplys are required for the reduction in the Tbx6 protein level, as observed in the mouse, and for proper formation of the anterior border of the Tbx6 domain in zebrafish embryos.

### Ripply can decrease Tbx6 protein level in zebrafish eggs

**Figure 5:**
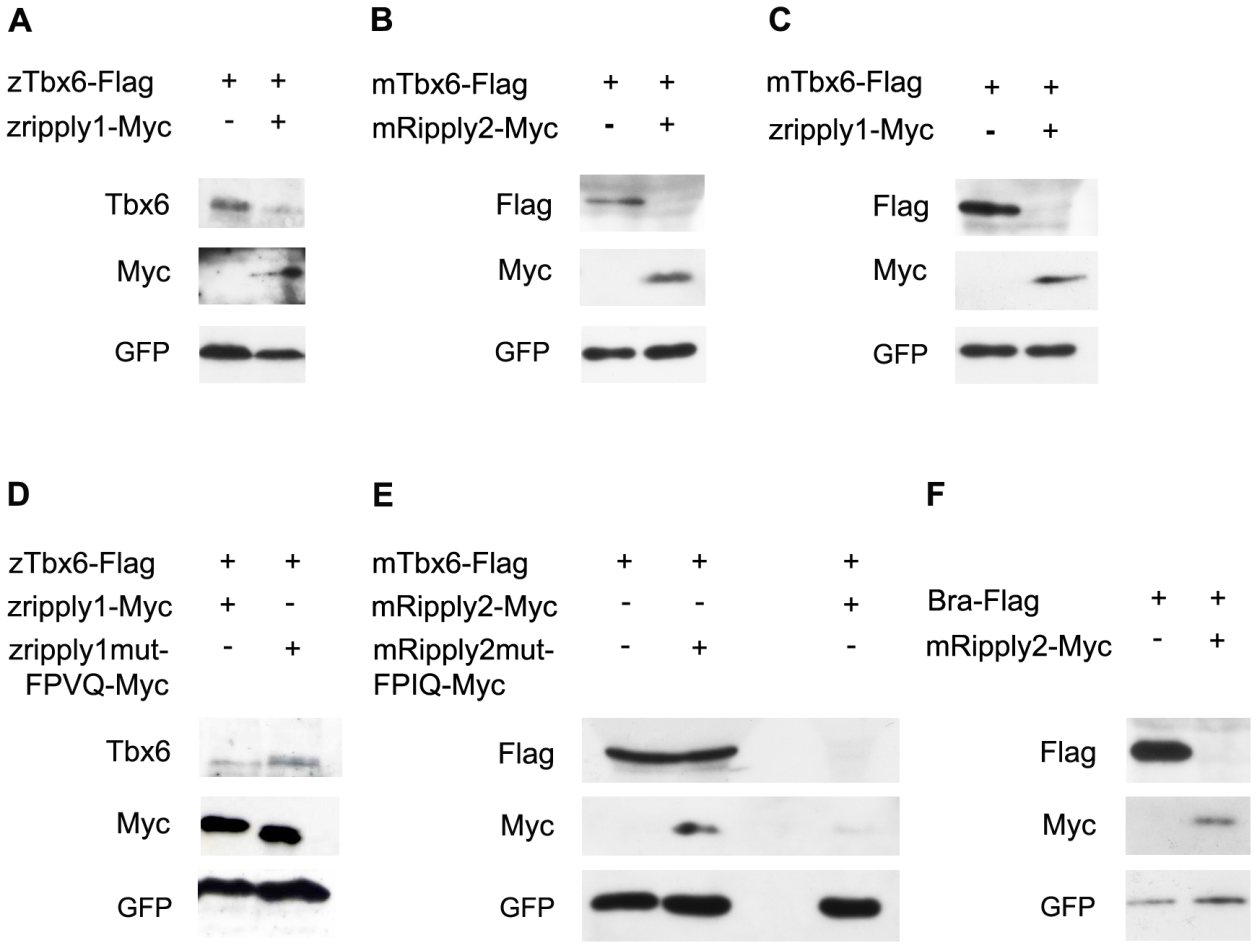
Ripply can reduce Tbx6 protein level. (A) Western blotting with proteins recovered from embryos injected with Flag-tagged zebrafish *tbx6 (ztbx6-Flag)* mRNA and Myc-tagged zebrafish *ripply1 (zripply1-Myc)* mRNA. Three hundred pg of *tbx6* mRNA with or without 300pg of zebrafish *ripply1-Myc* mRNA were injected into zebrafish eggs at 1 cell stage. (B, C) Similar experiments shown in (A) were performed with 150pg of Flag-tagged mouse *Tbx6* (*mTbx6-Flag*) mRNA and 50pg of mouse Myc-tagged *Ripply2 (mRipply2-Myc)* mRNA (B) or 120pg of zebrafish *ripply1-Myc* mRNA (C). Mouse Tbx6 proteins reduced when injected together with mouse *Ripply2* mRNA (B) and zebrafish *ripply1* (C). (D) Similar experiments shown in (A) were performed with 300pg of zebrafish *tbx6-Flag* mRNA, and 300pg of wild-type or FPVQ-mutated form of zebrafish *ripply1-6Myc* mRNA. In FPVQ-mutated form, this 4 amino acid stretch was replaced with four alanines as previously described [26]. (E) Similar experiments shown in (A) were performed with 150pg of mouse *Tbx6-Flag* mRNA and 50pg of wild-type or FPIQ-mutated form of mouse *Ripply2-Myc* mRNA. The FPIQ stretch in the mouse Ripply2 exists at the corresponding position to the FPVQ in the zebrafish Ripply1. In FPVQ-mutated form, this 4 amino acid stretch was replaced with four alanines. When co-injected with the mutated forms of zebrafish *ripply1* or mouse *Ripply2*, the reduction of Tbx6 proteins was canceled. (F) Similar experiments shown in (A) were performed with 50pg of mouse Flag-tagged *Brachyury (T)* mRNA and 50pg of mouse *Ripply2-Myc* mRNA. Mouse *Ripply2* also reduced mouse Brachyury protein level. As internal controls to validate the consistency between injection experiments, 100pg (B, C, E, F) or 200pg (A, D) of *GFP* mRNA was also injected and its expression was examined.

Next, we asked the molecular mechanism by which the anterior border of the Tbx6 domain was established in the PSM. In the mouse, *Mesp2* is one of the key molecules involved in this establishment, because a newly formed border of the Tbx6 domain is established nearby the caudal border of the *Mesp2* expression domain [14]. Furthermore, the anterior border of the Tbx6 domain is anteriorly expanded in *Mesp2*-deficient mouse embryos. These results indicate the requirement of *Mesp2* in the proper positioning of the Tbx6 domain [14]. Similarly, *Ripply1* and *Ripply2* are also required for this positioning, because *Ripply1* and *Ripply2* double-deficient embryos also exhibited anterior expansion of the Tbx6 domain [20]. Because expression of *Ripply1* and *Ripply2* is lost in the PSM in *Mesp2* mutant embryos [20], it seems likely that the loss of Ripplys' expression is a more direct cause for anterior expansion of the Tbx6 domain in *Mesp2*-deficient embryos. Furthermore, *Ripply1/Ripply2* double-mutant embryos rather exhibited increased expression of *Mesp2* although the anterior border of the Tbx6 domain was also expanded. Thus, *Mesp2* expression itself was not sufficient for elimination of Tbx6 proteins, which is required for the anterior positioning of the Tbx6 domain. Rather, *Ripply1* and *Ripply2* appear to play a role downstream or parallel to *Mesp2* in the anterior positioning of the Tbx6 domain. Therefore, we next examined whether Ripply could actually reduce the Tbx6 protein level. First, we used the COS7 cell line for this analysis, but failed to detect a Ripply-dependent reduction in the level of mouse Tbx6 proteins (data not shown). Next, we used the zebrafish egg as an assay system to examine whether a reduction in the Tbx6 protein level could be detected by injecting mouse or zebrafish *Tbx6* mRNA along with *Ripply* mRNA into zebrafish eggs (Figure 5). The amount of zebrafish Tbx6 protein was severely decreased by injection of zebrafish *ripply1* mRNA, indicating that Ripply possessed strong activity to reduce the Tbx6 protein level (Figure 5A). Similarly, mouse *Ripply2* mRNA also decreased the mouse Tbx6 protein level (Figure 5B), as did zebrafish *ripply1* mRNA (Figure 5C). Thus, the ability of Ripplys to reduce Tbx6 protein level is conserved between mouse and zebrafish. Of note, these effects by Ripplys were canceled when a FPVQ in zebrafish Ripply1 or its corresponding amino acid stretch in mouse Ripply2, FPIQ, both of which are amino-acid sequences essential for physical association with Tbx6 [26], Supplemental Figure S4), was deleted (Figures 5D and 5E). Thus, Ripply reduced the Tbx6 protein level probably through a direct protein-to-protein interaction. In addition to that of Tbx6, the protein level of another T-box factor, mouse Brachyury, was decreased by Ripply2 (Figure 5F), indicating that Ripply can reduce the level of several T-box proteins.

### Regulation of *ripply* expression in zebrafish embryos

**Figure 6:**
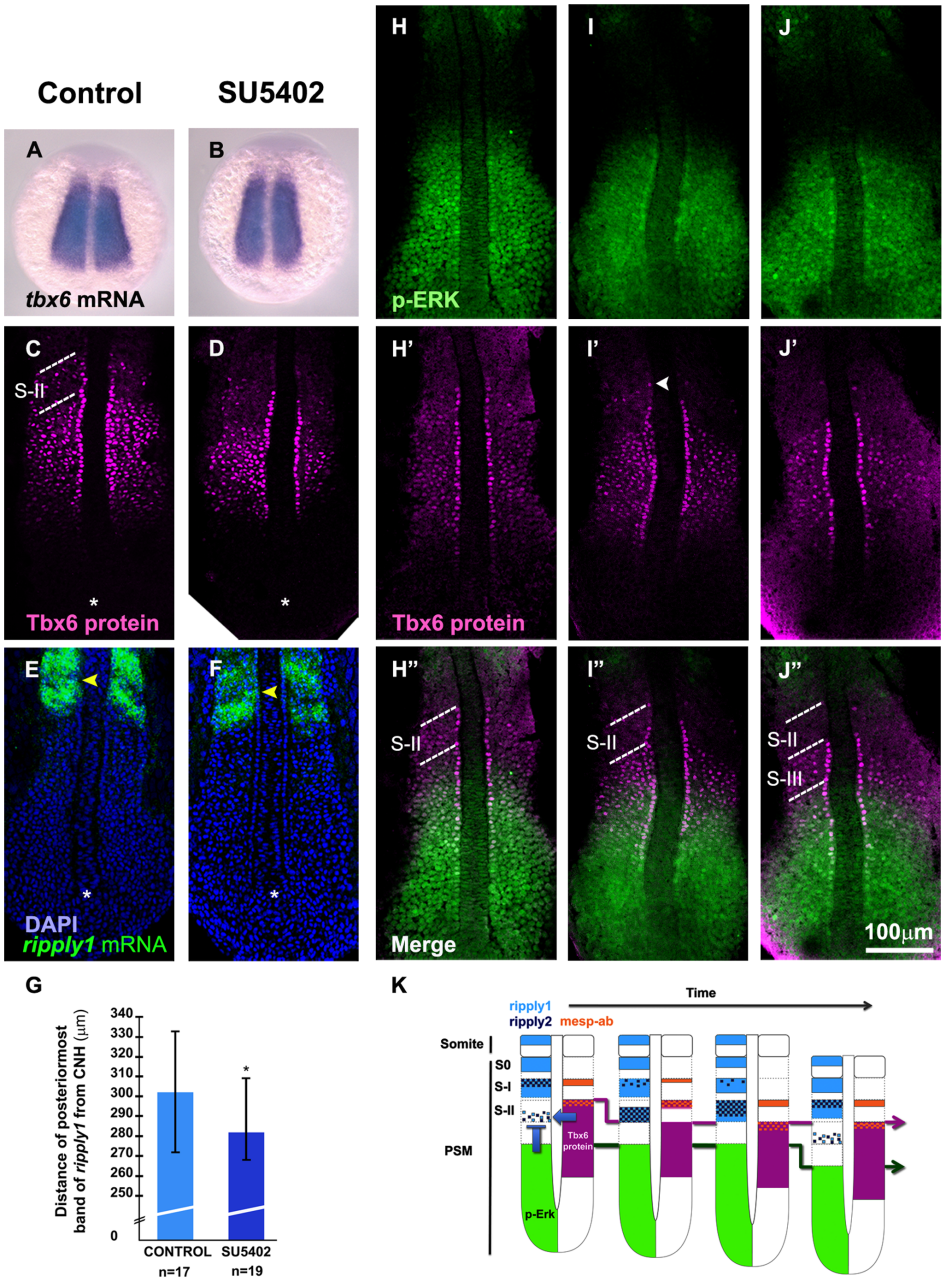
FGF signaling is required for *ripply* suppression. (A-F) Effect of SU5402, a chemical inhibitor against FGF signaling, on *tbx6* mRNA (A, B), Tbx6 protein (C, D) and *ripply1* mRNA (E, F) patterns in embryos at the 8 somite stage. Control embryos treated with DMSO (A, C, E) and embryos treated with SU5402 (B, D, F) are shown. While *tbx6* mRNA expression was unchanged (A, B), the anterior border of the Tbx6 domain was moved posteriorly in SU5402 treated embryos when compared with control embryos at the same phase of the segmentation cycle (C, D). Note that both of these embryos are at the stage when Tbx6 proteins just started to be eliminated in the anterior domain. A total of 15 number set of embryos were observed each for A and B, and all of the treated embryos did not show any change in *tbx6* mRNA expression pattern when compared to control embryos. Another 32 number set of embryos were treated with SU5402 and examined for Tbx6 protein, where, about 87% of the embryos showed posterior shift of anterior domain of Tbx6 protein when compared to control embryos. (E, F) *ripply1* expression is initiated earlier (yellow arrowheads) in SU5402 treated embryos (F) when compared to control ones (E) at the same phase of the segmentation cycle. Asterisk indicates the position of the chordo neural hinge (CNH). (G) The distance of the anterior border of the posteriormost expression of *ripply1* from the CNH in SU5402 treated embryos was significantly shorter than the control embryos; * *p<0.05* (*n* = 17 for control embryos and *n* = 19 for SU5402 treated embryos). Error bars indicate standard deviation. (H-J) Spatial distribution of FGF signaling was examined during a segmentation cycle in comparison with Tbx6 protein domain at the 8 somite stage. FGF signaling was monitored by staining with anti-phosphorylated Erk antibody. The upper band is indicated by an arrowhead in white. (K) A schematic representation of spatial patterns of Tbx6 and p-Erk domains with *ripply1*, *ripply2*, and *mesp-ab* expressions during single segmentation cycle. Expression of *ripply1* and *ripply2* is initially activated in the high Tbx6/low FGF signaling region. These activated Ripplys appear to suppress Tbx6 protein resulting in formation of a new anterior border of the Tbx6 core domain and the upper band. Then, expansion of *ripply* expression domain causes elimination of the upper band of Tbx6 protein. On the other hand, *mesp-ab* expression is activated at the anterior border of the Tbx6 domain, and remained at the same position. The dotted lines indicate S-II (C, H″, I″) and, S-II and S-III (J″) regions.

Because *ripply1* and *ripply2* were necessary and sufficient for reducing the level of Tbx6 proteins, an understanding of the regulation of their expression would be important for also understanding the mechanism of the boundary formation of somites. Tbx6 is a positive regulator in this regulation because the expression of *ripply1* and *ripply2* is lost in *tbx6/fss* mutant zebrafish embryos [23]. In contrast, since the Tbx6 domain is posteriorly shifted in mouse embryos defective in the FGF receptor 1 [14], it seems plausible to consider that FGF signaling may negatively regulate *ripplys*' expression in the PSM. To test this possibility, we examined Tbx6 domain and *ripply1* expression in zebrafish embryos treated with SU5402, a chemical inhibitor of FGF signaling. As predicted, SU5402 treatment caused a posterior shift in the Tbx6 domain in zebrafish embryos (Figures 6C and 6D), although the expression of *tbx6* mRNA was not obviously changed by this treatment (Figures 6A and 6B). Furthermore, SU5402 treatment also caused a posterior shift in *ripply1* expression at 2 hours after the treatment (Figures 6E, 6F, 6G, and Figure S5). Thus, this inhibition hastened the onset of *ripply1*, indicating FGF signaling was required for suppression of *ripply* expression in the PSM.

Recently, the anterior border of FGF activity was shown to shift posteriorly in a stepwise manner during a single segmentation cycle in zebrafish embryos [33]. Because this border corresponds to future somite boundary, it was proposed that the positioning of prospective somite boundary is already defined at this border of FGF signaling. If this is the case, it should be interesting to understand the process in which this stepwise shift of FGF signaling border leads to the stepwise shift of the Tbx6 domain, especially in terms of regulation of *ripply* expression. Thus, we next examined the spatio-temporal activation of FGF signaling, compared with the position of the Tbx6 domain (Figures 6H-6J) and *ripply* expression as well. The anterior border of FGF signaling, monitored with anti-phosphorylated Erk antibody [33], was positioned posterior to that of the Tbx6 domain in all of embryos examined. As far as our observation, the gap between these 2 borders changed almost within 1 to 2 segment lengths during a segmentation cycle. Comparing these results with the expression of *ripply1* and *ripply2* shown in Figure 3, we concluded that the initial or most posterior expression of *ripply1* and *ripply2* was observed in this gap region (Figures 3E-3G, Figures 6H-6K Figure S2), indicating that expression of the *ripply* genes was primarily established within the region where the level of Tbx6 was high and that of FGF signaling was low. Thus, a state with high Tbx6 protein and low FGF signaling is likely to be requisite for *ripply* expression; and periodical activation of *ripply* genes in the high Tbx6/low FGF signaling zone appears to have caused elimination of Tbx6 proteins in this zone and subsequent positioning of the intersomitic boundary.

## Discussion

### Mechanism of Ripply-mediated reduction in Tbx6 protein level

Creation of a discrete border of Tbx6 proteins in the anterior PSM was first reported in the mouse [16]. Since the expression of *Mesp2* requires Tbx6, this border accordingly defines the expression domain of *Mesp2*, which specifies the rostral side of a somite [17]. Therefore, the creation of the anterior border of the Tbx6 domain has been considered to be a crucial process in the positioning of the segmentation boundaries of somites. Here, using zebrafish eggs as an assay system, we showed that both mouse and zebrafish Ripply could act in reducing the Tbx6 protein level. We also showed that physical interaction between Tbx6 and Ripply appears to be required for this reduction, because a mutant form of Ripply that could not interact with Tbx6 was not able to cause this reduction.

Interestingly, the reduction in the Tbx6 protein level in the PSM appeared to be regulated in a ubiquitin-proteasome-dependent manner, because mouse embryos treated with chemical inhibitor of proteasome, MG132, exhibit anterior expansion of the Tbx6 domain [14]. Thus, it seems highly plausible that a ubiquitin-proteasome machinery is involved in the Ripply-mediated reduction of Tbx6 protein level. Given that Ripply family proteins are relatively small, consisting of about 100 amino acids [23], and do not possess similarity to any component of ubiquitin-proteasome machineries known to us, it is likely that some other component, directly or indirectly involved in the ubiquitin-proteasome machinery, may associate with the Tbx6-Ripply protein complex. At present, it is uncertain if such a molecule is actually involved in the determination of the Tbx6 domain; but further extensive analysis, for instance, screening and identification of Ripply-associated molecules, should make it clear.

### Mechanism of boundary positioning and rostro-caudal patterning in zebrafish somitogenesis

Previously, 2 different functions of Ripply were proposed with respect to the regulation of Tbx6 during somite segmentation. One of them is a reduction in the Tbx6 protein level [20]; and the other, suppression of the transcriptional activity of Tbx6 by recruiting the co-repressor Groucho/TLE to Tbx6 [23]–[27]. In the mouse, we showed that the level of the Tbx6 protein, but not that of its mRNA, is specifically affected in *Ripply1/Ripply2* double mutants [20]. A mathematical modeling based on this finding strongly suggests that Ripply's role in Tbx6 expression can be more suitably explained by its function in protein reduction rather than that in transcriptional regulation [20]. On the other hand, it had been unclear until now whether Ripply may play the same role in the somite segmentation in another animal such as the zebrafish. Rather, our previous studies with culture cells showed a function of zebrafish Ripply in transcriptional regulation of Tbx6.

In this study, by generating anti-zebrafish Tbx6 antibody, we succeeded in showing that a presumptive somite boundary was created at the anterior border of the Tbx6 domain. Moreover, analysis with *ripply1* and *ripply2* double-deficient embryos showed that Tbx6 protein level was increased, indicating that *ripply1* and *ripply2* normally reduce Tbx6 protein in the anterior PSM. These results strongly support the idea that reducing Tbx6 protein expression may be the major function of Ripply even in the zebrafish, although we cannot exclude the other possibility that Ripply-mediated transcriptional regulation may also play a role.

Given that Ripply is a regulator that defines the anterior border of the Tbx6 domain in both the mouse and the zebrafish, one of the critical processes in the positioning of the somite boundary should be the regulation of *Ripply* expression. In the mouse, the expression of *Ripply1* and *Ripply2* in the PSM is dependent on Mesp2, because expression of these *Ripply*s is lost in *Mesp2* null-mutant embryos [18], [20]. At present, it is uncertain whether this regulation between Ripply and Mesp is conserved even in zebrafish. Especially, since the period of segmentation is shorter in zebrafish somitogenesis (20 to 30 min) than in the mouse one (120 min), zebrafish may require a more speedy interaction for the generation of each boundary.

Another important point for understanding the mechanism of the boundary positioning is how the temporal information created by the oscillation affects the timing of *ripply* expression and Tbx6 border formation. In the mouse, the combination of the oscillatory changes in both Notch and FGF signalings determines the onset of *Mesp2* expression in the anterior PSM [12]. Since the activation of *Ripply1* and *Ripply2* expression and subsequent definition of the Tbx6 protein border is dependent on this *Mesp2* expression in the mouse, the Mesp2/Ripply/Tbx6-mediated machinery converts the oscillation into the boundary positioning [20]. In the zebrafish, in addition to the uncertainty of involvement of *mesp* genes, Notch signaling does not seem to be required for induction of *ripply* expression but is needed for proper patterning of it, since *ripply1* and *ripply2* are still expressed in Notch-defective embryos in spite of impaired pattern of expression [23]. On the other hand, we showed that FGF signaling negatively regulated expression of *ripply1* and *ripply2* in the zebrafish, similarly as in the mouse. Expression of *ripply1* and *ripply2* was induced in the high Tbx6 protein/low FGF signaling zone. Because the anterior border of FGF activity is periodically shifted in a step-wise fashion being consistent with the segmentation cycle in the zebrafish [33], the periodical retreat of the FGF border may regulate the timing and positioning of *ripply1* and *ripply2* expression. This periodical activation of *ripply* expression and subsequent interaction between Ripply and Tbx6 proteins appear to result in periodical creation of the anterior border of the Tbx6 protein domain. On the other hand, given that *ripply1* and *ripply2* expression shift from caudal to rostral part within a somite (Figure 6K), it seems also plausible that some oscillatory molecule, but not Notch and FGF signalings, regulated the expression of *ripply1* and *ripply2* in this zone in the zebrafish. Further extensive study should reveal the similarity and/or diversity in the mechanism underlying the positioning of intersomitic boundary between zebrafish and mouse and identify the core and conserved process resulting in the boundary positioning.

Finally, we would like to note that the pattern of Tbx6 proteins, which we observed in this study, may provide a clue for understanding the mechanism of the rostro-caudal patterning of a somite. In addition to the lack of somite boundaries, *tbx6/fss* zebrafish mutants display caudalization of the somites. However, this caudalization phenotype has not yet been well explained because *tbx6* mRNA is widely expressed in the anterior PSM. Interestingly, we found that Tbx6 proteins remain for a while at the rostral side of a presumptive somite, forming the “upper band”. Given that *mesp-ba* expression is dependent on Tbx6 even in zebrafish, the persistent Tbx6 proteins seem to be important for rostralization, because it is likely that their presence results in rostral-specific enhancement of *mesp-ba* expression.

## Supporting Information

Figure S1 **Specificity for antibody against zebrafish Tbx6.** The newly generated antibodies against zebrafish Tbx6 were tested for reactivity and specificity by western blotting. Cell lysates prepared from 293T cells expressing zebrafish Tbx6 tagged with Flag peptide at C terminus were loaded on SDS gel. Detection was achieved with both antisera #1 and #2, and also with antibody against Flag tag at the appropriate size. * indicates the detected zTbx6 protein bands.
(TIF)

Figure S2 **Expression of** ***mesp-ba*** **and Tbx6 at the prospective segmentation boundary.** Expression of *mesp-ba* in relation to the Tbx6 protein expression during different phases of somite segmentation. (A-A″) At the phase where the Tbx6 protein is expressed as a long core domain, the *mesp-ba* expresses as the three band pattern with the posteriormost band coinciding with the anterior border of Tbx6 at S-II. (B-B″) When the anterior region of Tbx6 starts to disappear, the posteriormost *mesp-ba* overlapped with the Tbx6 upper band, while the anteriormost band slowly disappears. (C-C″) The upper band of Tbx6 disappears but the core domain was shorter than in (A). At this phase, the *mesp-ba* expressed at the Tbx6 border does not yet emerge. Arrowhead (white) indicates the upper band. The S-II and S-III regions are shown by dotted lines.
(TIF)

Figure S3 **Expression of zebrafish** ***ripply2*** **and Tbx6 protein.** (A-A″) The expression of *ripply2* mRNA was initiated at the anterior region of the Tbx6 domain when the core domain was longer (B-B″). Accordingly to the increase in *ripply2* expression, Tbx6 proteins were eliminated in *ripply2* positive area, resulting in gap between the upper band and the core domain of the Tbx6 expression. (C-C″) Finally, when the Tbx6 anterior region was completely eliminated, *ripply2* was strongly expressed in S-II region. The S-II and S-III regions are marked by dotted lines. The phases shown in A, B and C are consistent with those in Fig.3. White arrowhead indicates the upper band of Tbx6 protein.
(TIF)

Figure S4 **Physical association between Tbx6 and Ripply.** Co-immunoprecipitation was conducted using various expression vectors. (A) 293T cells were transfected with zebrafish ztbx6-Flag, zRipply1-6Myc, or zRipply1-mutFPVQ-6Myc and co-immunoprecipitated with anti-Flag antibody and western blotting with either anti-Myc or anti-Flag antibodies. The zRipply1-6Myc co-immunoprecipitated with ztbx6-Flag, but the mutated zRipply1-mutFPVQ-6Myc did not. (B) Similarly, Cos 7 cells were also transfected with mouse mTbx6-Flag, in addition with mRipply2-Myc, or mRipply2-mutFPIQ-Myc followed by co-immunoprecipitation with anti-Flag antibody. mRipply2-Myc, but not mRipply2-mutFPIQ-Myc, co-mmunoprecipitated with mTbx6-Flag. The proteins were detected with the specified antibodies.
(TIF)

Figure S5 **The position of future somite boundary is pre-determined by FGF.** Embryos were treated with SU5402, an FGF inhibitor, (B, D) and compared with control embryos (A, C). Treatment was initiated at 2ss for 8 min and immediately after treatment, the embryos were either fixed with 4%PFA at 4°C overnight (C, D) or thoroughly washed and incubated at 28.5°C, then fixed at 6ss with 4%PFA at 4°C overnight (A, B). Note that no significant changes were observed in the Tbx6 protein expression in SU5402 treated embryos that were fixed immediately (D), when compared to the control embryos (C). In contrast, after four to five somite cycles, the anterior border of the Tbx6 protein showed posterior shift in the SU5402 treated embryos (B), unlike the control embryos (A) when compared at the same phase. * indicates the chordo neural hinge (CNH). The position of the S-II region is highlighted in dotted lines. Yellow arrowheads indicate the upper band of the Tbx6 protein.
(TIF)
