# Supplementary figures and images for: Tbx Protein Level Critical for Clock-Mediated Somite Positioning Is Regulated through Interaction between Tbx and Ripply
Source: PLoS One. 2014 Sep 26;9(9):e107928. doi: 10.1371/journal.pone.0107928 (PMC4178057; doi:10.1371/journal.pone.0107928)

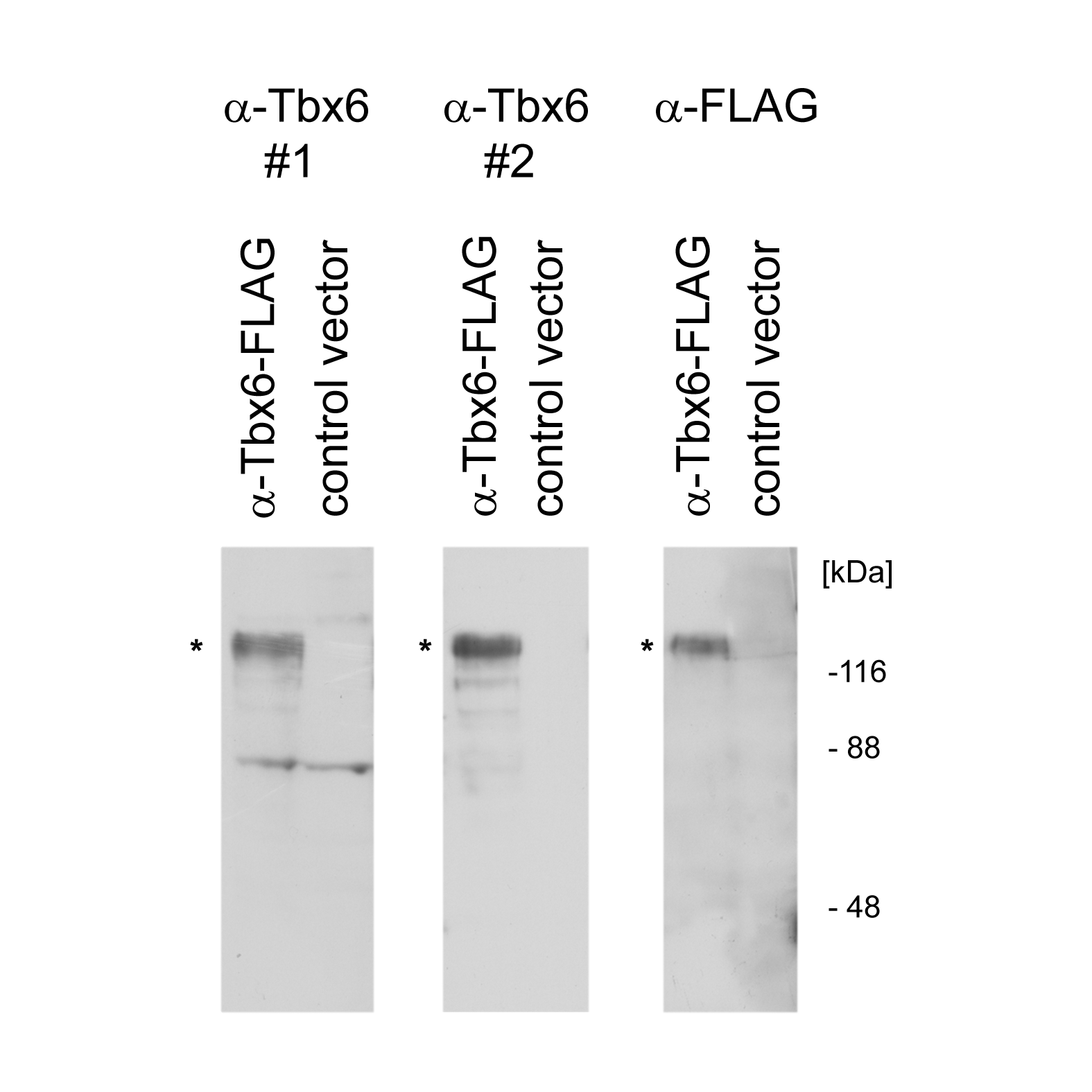

Supplement: Figure S1 — Specificity for antibody against zebrafish Tbx6. The newly generated antibodies against zebrafish Tbx6 were tested for reactivity and specificity by western blotting. Cell lysates prepared from 293T cells expressing zebrafish Tbx6 tagged with Flag peptide at C terminus were loaded on SDS gel. Detection was achieved with both antisera #1 and #2, and also with antibody against Flag tag at the appropriate size. * indicates the detected zTbx6 protein bands. (TIF) [file pone.0107928.s001.tif]

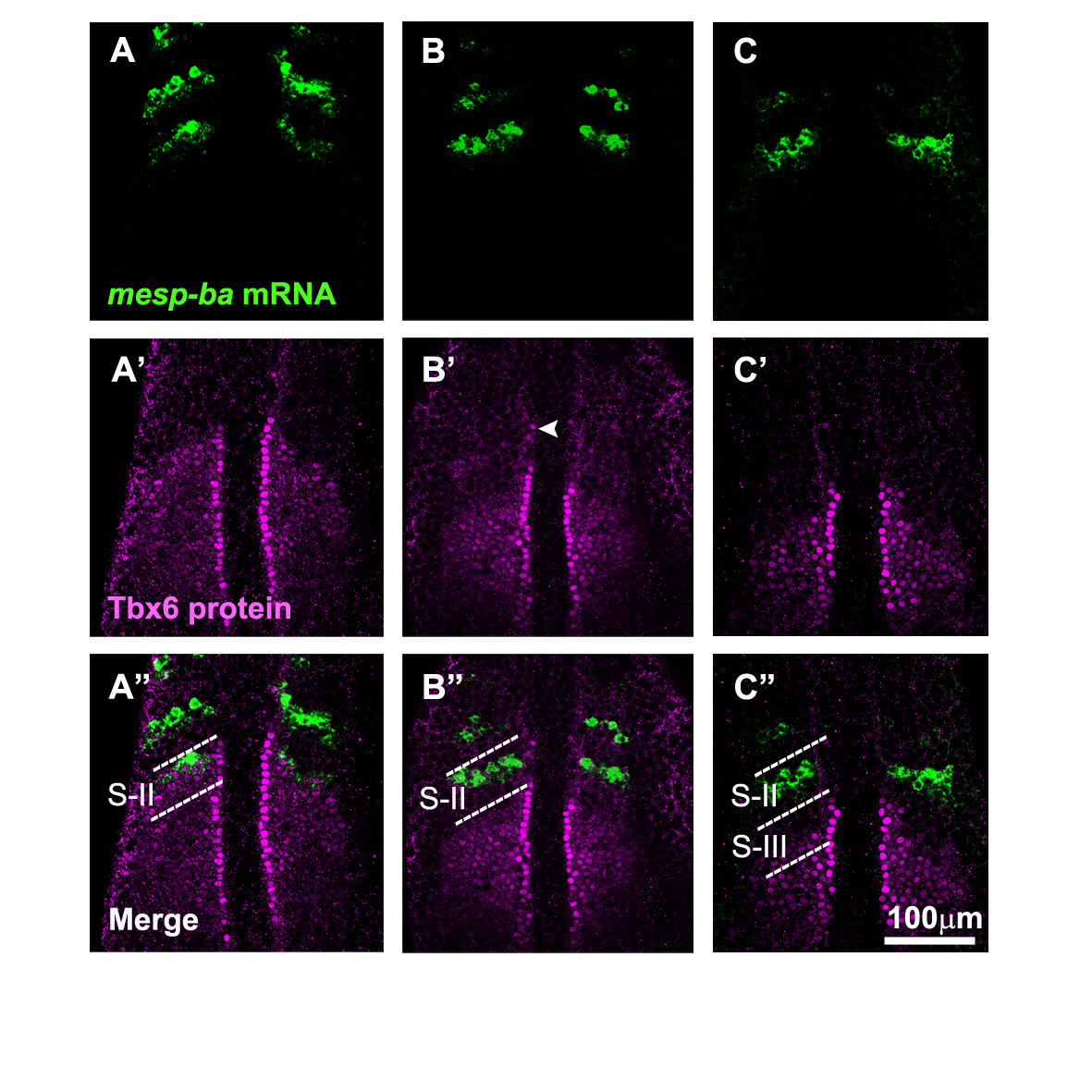

Supplement: Figure S2 — Expression of mesp-ba and Tbx6 at the prospective segmentation boundary. Expression of mesp-ba in relation to the Tbx6 protein expression during different phases of somite segmentation. (A-A″) At the phase where the Tbx6 protein is expressed as a long core domain, the mesp-ba expresses as the three band pattern with the posteriormost band coinciding with the anterior border of Tbx6 at S-II. (B-B″) When the anterior region of Tbx6 starts to disappear, the posteriormost mesp-ba overlapped with the Tbx6 upper band, while the anteriormost band slowly disappears. (C-C″) The upper band of Tbx6 disappears but the core domain was shorter than in (A). At this phase, the mesp-ba expressed at the Tbx6 border does not yet emerge. Arrowhead (white) indicates the upper band. The S-II and S-III regions are shown by dotted lines. (TIF) [file pone.0107928.s002.tif]

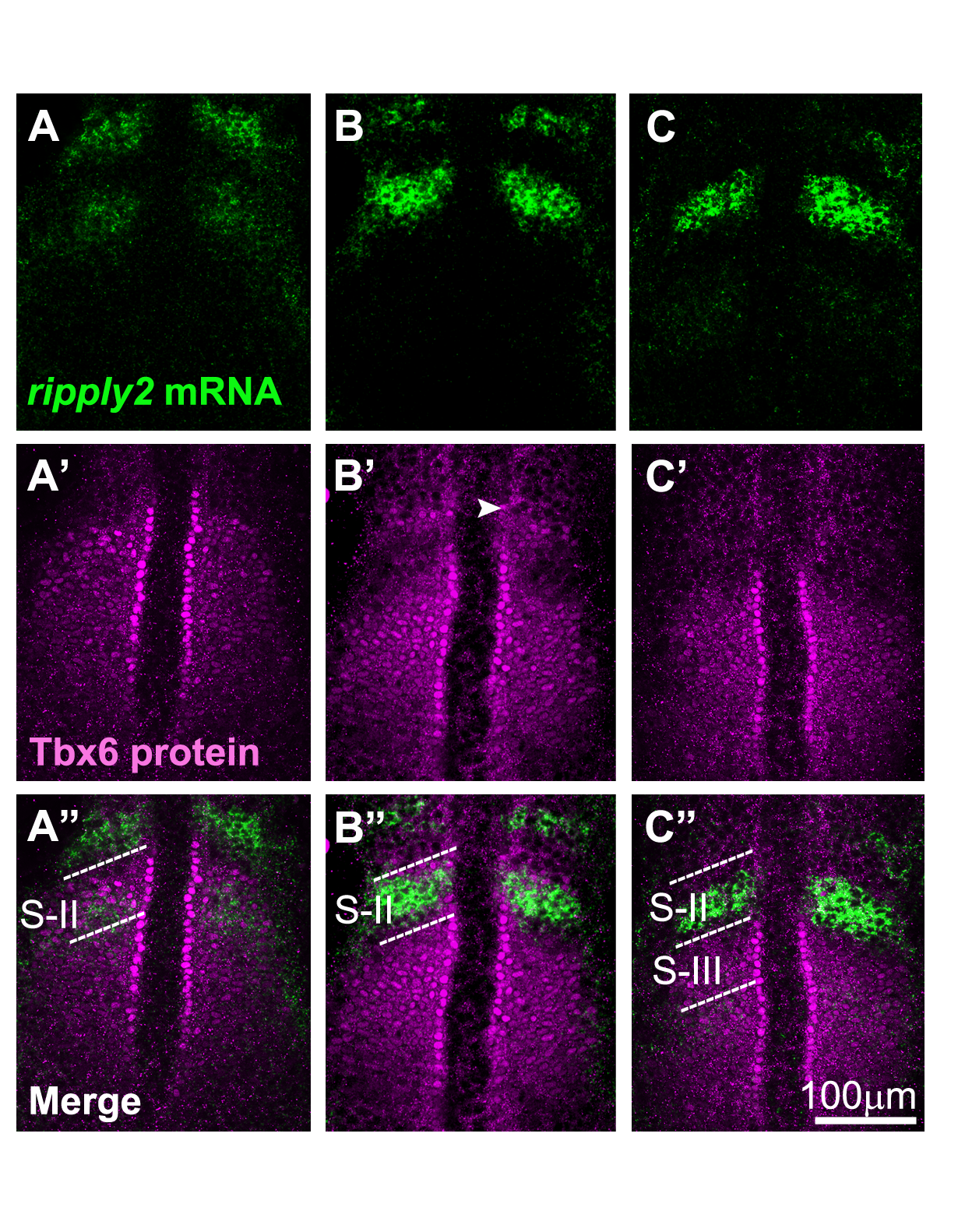

Supplement: Figure S3 — Expression of zebrafish ripply2 and Tbx6 protein. (A-A″) The expression of ripply2 mRNA was initiated at the anterior region of the Tbx6 domain when the core domain was longer (B-B″). Accordingly to the increase in ripply2 expression, Tbx6 proteins were eliminated in ripply2 positive area, resulting in gap between the upper band and the core domain of the Tbx6 expression. (C-C″) Finally, when the Tbx6 anterior region was completely eliminated, ripply2 was strongly expressed in S-II region. The S-II and S-III regions are marked by dotted lines. The phases shown in A, B and C are consistent with those in Fig.3. White arrowhead indicates the upper band of Tbx6 protein. (TIF) [file pone.0107928.s003.tif]

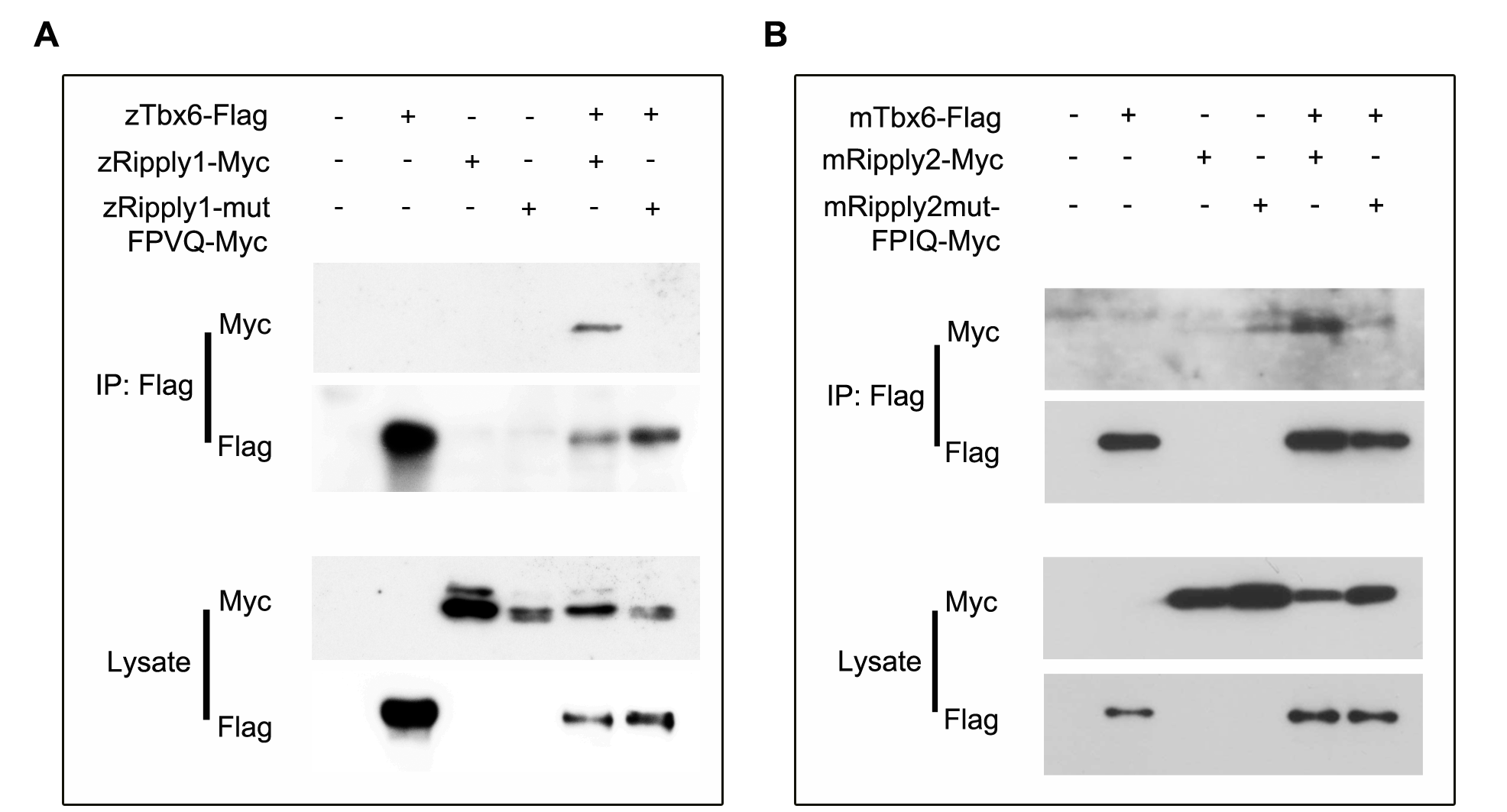

Supplement: Figure S4 — Physical association between Tbx6 and Ripply. Co-immunoprecipitation was conducted using various expression vectors. (A) 293T cells were transfected with zebrafish ztbx6-Flag, zRipply1-6Myc, or zRipply1-mutFPVQ-6Myc and co-immunoprecipitated with anti-Flag antibody and western blotting with either anti-Myc or anti-Flag antibodies. The zRipply1-6Myc co-immunoprecipitated with ztbx6-Flag, but the mutated zRipply1-mutFPVQ-6Myc did not. (B) Similarly, Cos 7 cells were also transfected with mouse mTbx6-Flag, in addition with mRipply2-Myc, or mRipply2-mutFPIQ-Myc followed by co-immunoprecipitation with anti-Flag antibody. mRipply2-Myc, but not mRipply2-mutFPIQ-Myc, co-mmunoprecipitated with mTbx6-Flag. The proteins were detected with the specified antibodies. (TIF) [file pone.0107928.s004.tif]

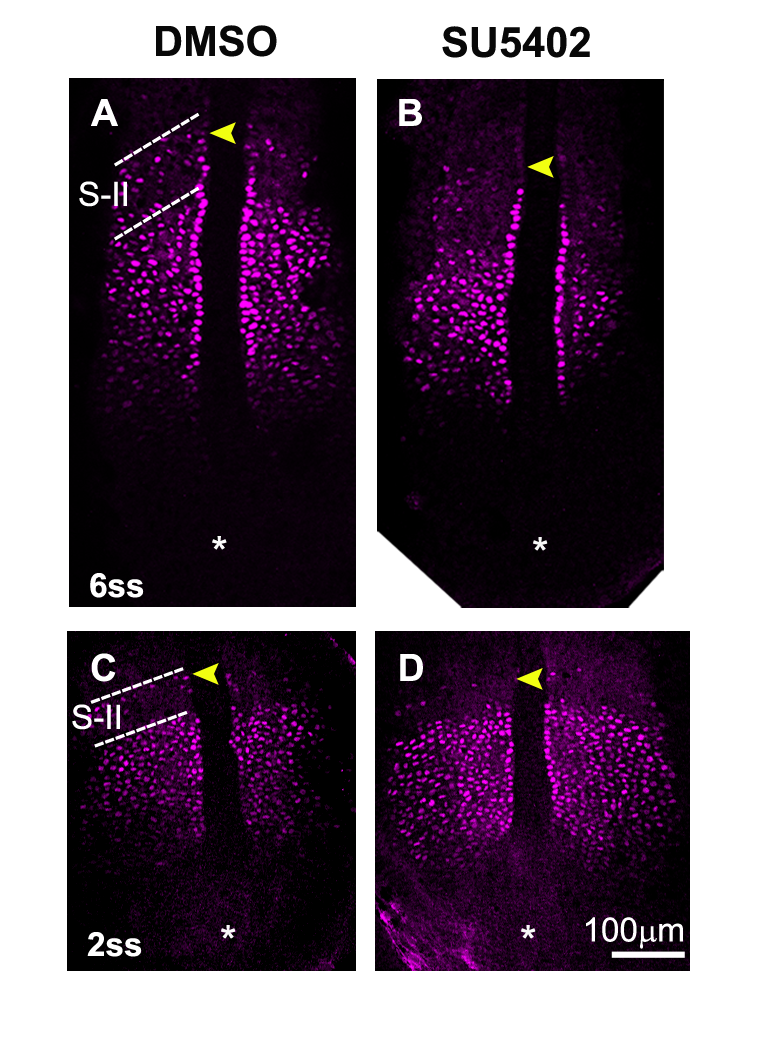

Supplement: Figure S5 — The position of future somite boundary is pre-determined by FGF. Embryos were treated with SU5402, an FGF inhibitor, (B, D) and compared with control embryos (A, C). Treatment was initiated at 2ss for 8 min and immediately after treatment, the embryos were either fixed with 4%PFA at 4°C overnight (C, D) or thoroughly washed and incubated at 28.5°C, then fixed at 6ss with 4%PFA at 4°C overnight (A, B). Note that no significant changes were observed in the Tbx6 protein expression in SU5402 treated embryos that were fixed immediately (D), when compared to the control embryos (C). In contrast, after four to five somite cycles, the anterior border of the Tbx6 protein showed posterior shift in the SU5402 treated embryos (B), unlike the control embryos (A) when compared at the same phase. * indicates the chordo neural hinge (CNH). The position of the S-II region is highlighted in dotted lines. Yellow arrowheads indicate the upper band of the Tbx6 protein. (TIF) [file pone.0107928.s005.tif]
